# Prairie Dog: Cuddly Pet or Trojan Horse?

**DOI:** 10.3201/eid1003.040045

**Published:** 2004-03

**Authors:** Abdu F. Azad

**Affiliations:** * University of Maryland School of Medicine, Baltimore, Maryland, USA

**Keywords:** prairie dogs, wild-caught animals, exotic pets, species jumping

**Figure:**
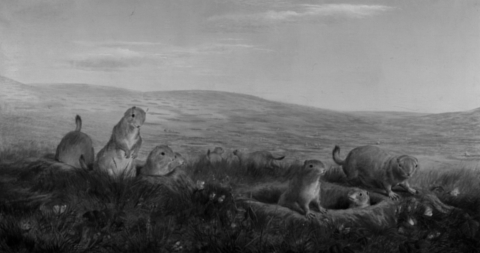
**William Jacob Hays (1830-1875). Prairie Dog Village (1860).** Oil on canvas (25 1/2 x 47 1/2). Collection of National Museum of Wildlife Art, Jackson Hole, WY

In this issue of Emerging Infectious Diseases, two articles analyze tularemia outbreaks, and one examines the pathologic features of monkeypox in commercially traded prairie dogs. While the prairie dog–associated tularemia outbreak and the first U.S. monkeypox outbreak highlight health risks to humans of the unregulated trade of wild-caught animals, they also raise broader issues. Exotic animal import and export have altered the composition of native fauna and flora throughout the world and have been associated with disease outbreaks. In addition, translocation of animals in some circumstances has provided the opportunity for pathogens to jump species and become established in native animal populations. Examples abound of intentionally or accidentally imported exotic species introducing new pathogens (e.g., rats introducing *Yersinia pestis*, the etiologic agent of plague, to the western United States) and allowing them to become established in native animal populations.

Avashia et al. (*1*) and Petersen et al. (*2*), analyzing the tularemia outbreak in commercially traded prairie dogs, underscore the difficulty of identifying existing infection in wild-caught animals, particularly by commercial collectors and distributors who trap prairie dogs from disease-endemic foci. The outbreak was predictable, considering the conditions in which the animals were kept. In retrospect, the prairie dog–associated monkeypox outbreak highlights the speed with which exotic rodent species, transported worldwide, allow virulent pathogens to jump species and be transmitted to humans. Often, infection with these pathogens is fatal for recipient animals, as is the case in prairie dogs infected with *Y. pestis* or *Francisella tularensis* (the etiologic agent of tularemia). The infected animals can also become “silent” carriers and serve as a new reservoir for the introduced pathogen. The overall outcome of a pathogen’s jumping species often remains unpredictable. Guarner et al. (*3*) investigated the pathologic features of monkeypox in prairie dogs to identify the route of viral transmission to humans. They found that viral transmission could occur through both respiratory and direct mucocutaneous exposure. In addition, prairie dogs may be an excellent model for assessing monkeypox viral transmission, pathogenesis, and new vaccines and treatments.

Recent incidents involving plague, tularemia, and monkeypox transmission to humans by pet prairie dogs are a wake-up call for better surveillance of wild-caught animals before they are sold internationally and imported into the United States. Relying on visual inspection to select healthy animals is virtually impossible, since wild-caught animals often do not exhibit signs of overt disease and may not appear sick during the early stages of infection with *Y. pestis* or *F. tularensis*. Prairie dog–associated monkeypox and tularemia, as discussed in Avashia et al., Petersen et al., and Guarner et al., highlight the need for more research, public education, regulatory guidelines for exotic animal husbandry practices, and possibly a ban on the sale of wild-caught animals. Although the examples in this issue relate to prairie dogs, they may well serve as harbingers of other emerging infections. In introducing seemingly harmless furry friends, the trade of exotic pets brings together species that have never encountered one another in nature, with unpredictable and sometimes tragic results.
